# A Review on Worldwide *Ephedra* History and Story: From Fossils to Natural Products Mass Spectroscopy Characterization and Biopharmacotherapy Potential

**DOI:** 10.1155/2020/1540638

**Published:** 2020-04-30

**Authors:** Khaoula Elhadef, Slim Smaoui, Mariam Fourati, Hajer Ben Hlima, Ahlem Chakchouk Mtibaa, Imen Sellem, Karim Ennouri, Lotfi Mellouli

**Affiliations:** ^1^Laboratory of Microorganisms and Biomolecules, Center of Biotechnology of Sfax, University of Sfax, Road of Sidi Mansour Km 6, P.O. Box 1177, Sfax 3018, Tunisia; ^2^Algae Biotechnology Unit, Biological Engineering Department, National School of Engineers of Sfax, University of Sfax, Sfax 3038, Tunisia

## Abstract

Growing worldwide, the genus *Ephedra* (family Ephedraceae) had a medicinal, ecological, and economic value. The extraordinary morphological diversity suggests that *Ephedra* was survivor of an ancient group, and its antiquity is also supported by fossil data. It has recently been suggested that *Ephedra* appeared 8–32 million years ago, and a few megafossils document its presence in the Early Cretaceous. Recently, the high analytical power provided by the new mass spectrometry (MS) instruments is making the characterization of *Ephedra* metabolites more feasible, such as ephedrine series. In this regard, the chemical compounds isolated from crude extracts, fractions, and few isolated compounds of *Ephedra* species were characterized by MS-based techniques (LC-MS, LC-ESI-MS, HPLC-PDA-ESI/MS, LC-DAD-ESI/MSn, LC/Orbitrap MS, etc.). Moreover, we carry out an exhaustive review of the scientific literature on biomedicine and pharmacotherapy (anticancer, antiproliferative, anti-inflammatory, antidiabetic, antihyperlipidemic, antiarthritic, and anti-influenza activities; proapoptotic and cytotoxic potential; and so on). Equally, antimicrobial and antioxidant activities were discussed. This review is focused on all these topics, along with current studies published in the last 5 years (2015–2019) providing in-depth information for readers.

## 1. Introduction

It is becoming increasingly clear that plants, ranging from across the plant kingdom, produce a unique diversity of secondary metabolites that can be exploited for the discovery of new drugs, bio-sourced materials, nutraceuticals, or cosmetics [1–5]. For finding new molecules, plant natural products are undoubtedly good sources of chemical diversity [6–10]. It is estimated that over 200,000 primary and secondary metabolites may be present in the plant kingdom [11–15]. Medicinal plant is the product of long-term medical practice worldwide, with the advantages of outstanding curative properties and less side effects [16–21]. Containing many natural products and their derivatives of therapeutic value, medicinal plants are considerate as main source of remedies able to protect human body against diseases.

As medicinal plant, enclosing over 50 species, *Ephedra* genus belongs to the family Ephedraceae which in turn represents one of three families in the order Gnetales [22–26]. *Ephedra* is common in cold and dry places in both the Old and the New Worlds; the Gnetaceae members live in warm and humid tropical/subtropical forests of Asia, Africa, and South America [27]. The shrubs, which reach approximately one meter in height, grow in semiarid and desert conditions in both hemispheres across six continents [25, 26]. Known as Ma Huang, *E. sinica* is one of the oldest medicinal herbs in traditional Chinese medicine [28–31]. *E. sinica* preparations have been used for over 5000 years as stimulants and as antiasthmatics and are traditionally used to treat cold, bronchial asthma, cough, fever, flu, headache, edema, and allergies [32, 33]. It can also be used to lose weight by increasing sweating and basal metabolism and by stimulating the central nervous system [34, 35]. Moreover, it has also been combined with cardiovascular drugs to treat cardiovascular diseases [36, 37]. For years, ephedrine series [(-)-ephedrine, (+)-pseudoephedrine, (-)-N-methylephedrine, (+)-N-methylpseudoephedrine, (-)-norephedrine, (+)-norpseudoephedrine] were considered to be the main *Ephedra* constituents [38, 39]. Nowadays, at the side of pharmacological effects, there has been considerable research on the phytochemistry and bioactivities of genus *Ephedra*, including their antibacterial and primarily antioxidant activity [40–44]. From the entire plant, a wide range of *Ephedra* natural products including alkaloids, tannins, saponins, proanthocyanidins, phenolic acids, flavonoids, and essential oils have been mentioned and the plants-derived polyphenols are of great importance for their biological and pharmacological potential [45–50].

However, these numbers may be underestimated since many metabolites have not been characterized yet and new publications appear continuously with numerous new structures. In the last two decades, there was a quick development of mass spectrometric techniques allowing analysis of *Ephedra* natural products. Mass spectrometry is currently one of the most versatile and sensitive instrumental methods applied to structural characterization of *Ephedra* metabolite [51–55].

Although the analysis of *Ephedra* natural products has been investigated for many years, there is not a review in the literature focusing on the great pharmacological and biological potential and applications of high-resolution mass spectrometry. In this review, we will provide information about these topics and their advances and applications in the last five years (2015–2019) that could be interesting for botanical, analytical chemistry, and natural products communities.

## *2. Ephedra* History Evolution

Containing approximately fifty species, the genus *Ephedra* (Family Ephedraceae) was distributed in arid and semiarid regions of Asia, Europe, northern Africa, southwestern North America, and South America [56–60]. In fact, *Ephedra* was distributed from the northern temperate zone (from the Canary Islands through the Mediterranean region and Central Asia to Shandong in China) to the arid regions of USA and Mexico, and to alpine area of the Andes in South America [26, 27,61–64]. This wide range is at least partially attributable to its effective dispersal syndromes.

Fossil evidence has been playing important roles in understanding early evolution of the *Ephedra* gnetophytes [65, 66]. The early evolution and diversification of the *Ephedra* have increasingly become clear because of recently reported macrofossils from the Early Cretaceous strata of Asia, Australia, Europe, and Americas [67, 68]. *Ephedra* macrofossils, especially female cones, provide a historical perspective for the early evolution, taxonomy, and biogeography of this genus. In this respect, by using molecular sequence data (rbcL) and assuming a constant rate of evolution calculated by landmark event calibration, the corresponding age of extant *Ephedra* was recently estimated to be 8–32 million years [69–72]. Macrofossils of female cones were found in the Early Cretaceous of South America [73, 74], Mongolia [75, 76], and adjacent Northeast China [60, 77–80]. Early *Ephedra* might have transformed bracts of female cones into vivid color to assist seed dispersal by birds, wind, or seed-caching rodents resulting in a wide intercontinental distribution [59, 81, 82].

On the other hand, phylogenetic analysis resulted in well-supported subgroups of *Ephedra* that correspond to geographical regions [69, 83–85] with African-Mediterranean species in a basal grade or clade and Asian species forming two well-supported clades [72, 86]. New World species are monophyletic and comprise a South American clade [72, 86] and a nonmonophyletic grade of North American species [86]. As reported by Crane [87], a possible origin of *Ephedra* in Africa is interesting as the diversity of ephedroid pollen grains is particularly high in Early Cretaceous palaeoequatorial regions of Africa and South America. In this context, African species constitute a basal grade or clade within *Ephedra* [72]: some of the basal species are limited to Africa (*E. altissima*); others have a broader distribution in the Old World, extending from Africa into Asia or southern Europe. In recent decades, various *Ephedra* and *Ephedra*-like meso- and macrofossils have been reported from the Early Cretaceous of South Europe, Northeast China [79], Mongolia [88], North America, and South America [25, 56, 89]. Seed mesofossils with in situ pollen were reported from the Early Cretaceous of North America [72] and Portugal (South Europe) [60].

## 3. *Ephedra* Extracts Phytochemical Content

From a chemical point of view, previous studies conducted on *Ephedra* showed that it contained different types of polyphenols, flavonoids, and anthocyanins [41, 42, 90–97]. For quantitative measurement, gallic acid (quercetin or catechin) and cyanidin-3-glucoside were used as standard compounds (references) to quantify total polyphenol, total flavonoid, and total anthocyanins content, respectively.

In a study carried out by Danciu et al. [90] on ethanolic extracts of the aerial part of *E. alata* Decne., an amount equal to 156.22 mg of gallic acid equivalents/g dry sample (mg GAE/g) was reported for total polyphenol (TPC). Jaradat et al. [91] have analyzed the phytochemical composition of *E. alata* Decne., by using water, MeOH, and EtOH for the extraction. The study reports that, when water was used, total polyphenols could not be detected in the extract, and the EtOH extract was 19.175 mg GAE/g. On the other hand, Alali et al. [92] and Nasar et al. [93] reported that water extracts of *E. alata* Decne. and *E. procera* C. A. Mey showed a TPC of 16.2 and 117.01 mg GAE/g, respectively.

Al-Rimawi et al. [41] analyzed extracts of *E. alata* Decne. collected from the southern part of the West Bank, Palestine. These authors used three different solvents, namely, water, 100% EtOH, and 80% EtOH, in order to observe which solvent leads to the highest amounts of total flavonoid contents (TFC). The results showed that TFC was higher in case of 100% EtOH (19.5 ± 0.3 mg catechin/g dry weight). Aghdasi et al. [42] studied the variation of TFC of Iranian *E. major* during May to October from Bojnoord. In fact, TFC exhibited a variation during sampling period and ranged from 4.63 to 8.4 mg QE/g. Mellado et al. [94] analyzed flavonoids in *E. chilensis* K Presl, a Chilean endemic plant. These authors reported significant differences in the CH_2_Cl_2_ extracts (*P* < 0.05) compared to the hexane and EtOH extracts. The total phenolic content in both CH_2_Cl_2_ extract and EtOH extract shows significant differences (*P* < 0.05) with the hexanoic extract. In the study of Al-Trad et al. [95], the butanolic extract from the stem of Jordanian *E. alte* had a phenolic content of 404.001 ± 5.53 mg/g gallic acid and flavonoids of 40.73 ± 6.59 mg/g quercetin [95].

Hegazy et al. [96] observed that the total anthocyanins content (TAC) of Saudi *E. foeminea*, collected from Shada Mountain, southwest Saudi Arabia, was 0.14 mg cy-3-glu/100 g. TAC of Lebanese *E. campylopoda* was detected in the MeOH extracts but not in EtOH and aqueous fractions [97].

## 4. Recent Applications of High-Resolution Mass Spectrometry for *Ephedra* Extract Characterization

Although the analysis of natural products from *Ephedra* species has been investigated for many years, there is not a review in the literature focusing on the great possibilities and applications of high-resolution mass spectrometry. This review is devoted to chemical identification using mass spectrometry as the most powerful technique of qualitative analysis. It is evident in the fact that the terms of “identification” and “mass spectrometry” occur together in more than a million scientific reports returned in the search results performed by Google Scholar engine [98]. The reason for the potency of MS is that it is superior to other analytical techniques in the combination of features, such as multianalytic property, sensitivity, selectivity, possibility of compounds identification by molecular mass or formula, and possibility of combining with chromatography.

**Figure 1:**
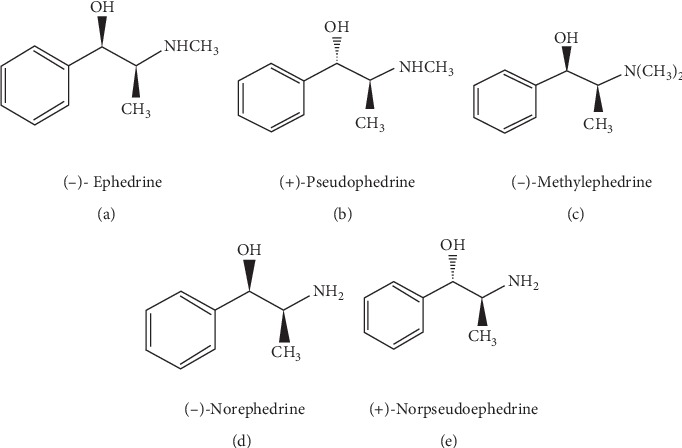
Chemical structures of ephedrine alkaloids.

The most prominent methods include MS/MS (tandem mass spectrometry); LC-ESI/MS/MSn (liquid chromatography–electrospray ionization/multistage mass spectrometry); LC-PDA (liquid chromatography coupled to photodiode array); HPLC-PDA-ESI/MS (high-performance liquid chromatography coupled to photodiode array and electrospray ionization mass spectrometric); LC-DAD-ESI/MSn (high-performance liquid chromatography with diode array detection coupled to tandem mass spectrometry analysis with electrospray ionization); and LC/Orbitrap MS (liquid chromatography Orbitrap Fusion Tribrid tandem mass spectrometry). In this section, we will provide information about this topic and its advances and applications in the last five years (2015–2019) that could be interesting for both the analytical chemistry and the natural products communities, from *Ephedra* species collected from the five continents of the world. The ephedrine alkaloids (Figure 1) are considered the active constituents of plants belonging to the genus *Ephedra*. (−)-Ephedrine is the major isomer; the minor alkaloids include (−)-norephedrine, (+)-norpseudoephedrine, (+)-pseudoephedrine, and (−)-methylephedrine [99].

As a summary, Table 1 shows some of the most commonly used methods to identify and quantify phenolic compounds (chromatographic conditions; mobile phase and gradient, quantification and detection, and analytical method) from *Ephedra* species extracts.

A phytochemical characterization of the hydroalcoholic (70% EtOH) extract of the aerial part of Tunisian *E. alata* Decne was reported by Danciu et al. [90]. Using LC-MS, detected individual polyphenols were gallic acid, protocatechuic acid, caffeic acid, coumaric acid, ferulic acid, rosmarinic acid, epicatechin, rutin, resveratrol, quercetin, and kaempferol. Under the same operating conditions, individual polyphenols were determined using two different C18 chromatographic columns: Adsorbosphere UHS C18 and EC 150/2 NUCLEODUR C18 Gravity SB. On both columns, identified compounds were rosmarinic acid (0.013 *µ*g/mg), resveratrol (0.223 *µ*g/mg), quercetin (2.63 *µ*g/mg), and kaempferol (15.55 *µ*g/mg). Caffeic acid and p-coumaric acid were identified in small quantities, respectively, at 0.014 and 0.05 *µ*g/mg. These compounds were identified only on the Adsorbosphere UHS C18 column, while epicatechin was identified on the NUCLEODUR C18 Gravity SB column. Using liquid chromatography–electrospray ionization–tandem mass spectrometry (LC-ESI-MS) analysis, Benabderrahim et al. [23] determined that Tunisian E. a*lata* ethanol (50%) extracts, collected from Saharan regions of South Tunisia, showed medium levels of quinic acid, p-coumaric acid, epicatechin, rutin, luteolin, and cirsilineol. Extracted by LC/PDA/ESI (−)/MS method, 24 phenolic compounds were found in the hydromethanol *E. alata* crude extract [45]. These phenolics were 10 phenolic acids (quinic acid, gallic acid, protocatechuic acid, chlorogenic acid, 4-O-caffeoylquinic acid, syringic acid, caffeic acid, p-coumaric acid, trans-ferulic acid, and trans-cinnamic acid); 5 flavones (apigenin, luteolin, cirsiliol, cirsilineol, and acacetin); 2 flavonols (quercetin and kaempferol); 2 flavan-3-ols ((+)-catechin and epicatechin); 2 flavonol glycosides (rutin and quercitrin); 2 flavone glycosides (apigenin-7-O-glucoside and naringin); and 1 flavanone (naringenin). For derivative fractions of MeOH crude extract, 19 phenolic compounds were detected in the EAc and BuOH, whereas 18 compounds were identified in the water and only 14 compounds were detected in the DCM. The EAc and the BuOH contained almost the same detected compounds (18 compounds among 19 identified). The main phenolic compounds in the EAc were (−)-epicatechin (5864.24 *μ*g/g dry extract (DE), quercetin-3-O-rhamnoside (3647.49 *μ*g/g DE), and (+)-catechin (3289.03 *μ*g/g DE). The principle phenolic compounds identified in the BuOH were quinic acid (847.79 *μ*g/g DE), naringin (682.98 *μ*g/g DE), (−)-epicatechin (363.15 *μ*g/*g* DE), quercetin-3-O-rhamnoside (309.59 *μ*g/g DE), (+)-catechin (201.0915 *μ*g/g DE), and apigenin-7-O-glucoside (99.51 *μ*g/g DE). In the aqueous fraction, the major phenolic compounds were quinic acid (2533.89 *μ*g/g DE), naringin (230.34 *μ*g/g DE), trans-cinnamic acid (71.87 *μ*g/g DE), and syringic acid (46.73 *μ*g/g DE). The most important phenolic compounds detected in DCM were trans-cinnamic acid, naringin, and trans-ferulic acid detected, respectively, at 2064.35, 1920.11, and 1406.31 *μ*g/g DE. Ziani et al. [100] reported that ten phenolic compounds, five isoflavones, and five flavones were characterized and performed by applying LC-DAD-ESI/MSn to three different extracts obtained from infusion, decoction, and maceration in hydroethanolic mixtures of Algerian *E. alata.*

Collected from Austria, dry herbs of *E. major*, *E. distachya* subsp. Helvetica, *E. monosperma*, *E. fragilis*, *E. foeminea*, *E. alata*, *E. altissima*, and *E. foliate* were used to separate and quantify ephedrine (E) and pseudoephedrine (PE) by UPLC-UV [102]. Using 5 ng as a limit of detection, among the analyzed species, *E* is the dominant alkaloid in *E. major*, *E. fragili*s, and *E. distachya* subsp. Helvetica. *E. monosperma* was the only species with a higher PE content. E and PE were not detected in *E. foeminea*.

Palestinian *E. alata* extracted with water, 80% ethanol, and 100% ethanol was rich in flavonoid glycosidic compounds. In fact, the full scanned LC-MS using the positive and negative electrospray ionization modes revealed the presence of luteolin-7-O-glucuronide flavonoid, myricetin 3-rhamnoside, and some other major polyphenolic compounds that share myricetin skeleton [41] Collected from Bojnoord (Iran), stems and seeds of *E. major* were soaked in 80% MeOH, and the amounts of ephedrine were determined by HPLC [42]. Data from HPLC analysis revealed that while root is depleted of ephedrine, the ephedrine amount in stem organ ranged from 1.50 to 2.12 mg/g dry weight. To assess the alkaloids present in Pakistani E. inte*rmedia*, the HPLC method was used for the quantitative analysis of ephedrine and pseudoephedrine [103]. This study showed that average alkaloid substance in *E. intermedia* was as follows: pseudoephedrine (0.209%, 0.238%, and 0.22%) and ephedrine (0.0538%, 0.0666%, and 0.0514%).

Hyuga et al. [107] described the preparation of an ephedrine alkaloids-free Japanese *Ephedra* herb extract (EFE) by ion-exchange column chromatograph. In this study, LC-PDA analysis of aqueous *Ephedra* herb extract and EFE was used. In fact, the *Ephedra* herb extract standard revealed the presence of ephedrine alkaloids (ephedrine, pseudoephedrine, norephedrine, and methylephedrine), 6-hydroxykynurenic acid, syringin, kaempferol 3-O-rhamnoside-7-O-glucoside, 6-methoxykynurenic acid, isovitexin 2″-O-rhamnoside, and cinnamic acid. However, ephedrine alkaloids, 6-hydroxykynurenic acid, and 6-methoxykynurenic acid were not present in the EFE chromatogram. Later, in 2018, Oshima et al. [108] analyzed *Ephedra* herb extracts grown in different habitats and collection years in Japan by liquid chromatography/high-resolution mass spectrometry (LC/HRMS). These authors detected two notable peaks common to each extract. These peaks were identified as vicenin-2 (1) and isovitexin 2″-*O*-rhamnoside (2). Quantitative analyses using the isocratic condition of LC/MS showed that the content percentages of 1 and 2 in EFE were 0.140–0.146% and 0.350–0.411%, respectively. Furthermore, Oshima et al. [108] analyzed apigenin (3), an aglycon common to 1 and 2. In 2016, Mei et al. [110] reported that the HPLC analysis determined five activity components of *Ephedra*-*Gypsum* extract. They were norephedrine (NE), norpseudoephedrine (NPE), ephedrine (E), pseudoephedrine (PE), and methylephedrine (ME) with contents of 0.143, 0.065, 1.723, 0.794, and 0.165 mg/g, respectively. A rapid hydrophilic interaction liquid chromatographic (HILIC) method has been developed and validated for simultaneous quantitative analysis of methylephedrine, ephedrine, and pseudoephedrine in Chinese *Ephedra* herb and its preparations [111]. The chromatographic method was validated for specificity, linearity and range, limit of detection and quantification, precision, stability, repeatability, and accuracy. The main parameters were specificity (peak purity match factors were >980), linearity (*r* > 0.9996), intra- and interday precisions (RSD%: 0.48–1.70 and 0.81∼1.86, respectively), and limit of detection and quantifications (29.49 and 98.31 ng/mL for methylephedrine; 47.74 and 159.1 ng/mL for ephedrine; 121.8 and 406.0 ng/mL for pseudoephedrine). On the other hand, two new compounds of phenylpropanoids, (*S*)–*N*-((1*R*,2*S*)-1-hydroxy-1-phenylpropan-2-yl)-5-oxopyrrolidine-2-carboxamide (1) and (3*R*)-3-O-*β*-d-glucopyranosyl-3-phenylpropanoic acid (2), were isolated from the Chinese *E. sinica* stems. Their structures were elucidated by in-depth examination of spectroscopic data, mainly including those from the 1D and 2D NMR technique, high-resolution electron spray ionization mass spectrum (HRESIMS) technique, and chemical method [112].

## 5. Biological Activities

### 5.1. Antioxidant Activity

The antioxidant activity of *Ephedra* was evaluated by cupric ion reducing capability in the presence of neocuproine: CUPRAC method, DPPH (2.2-diphenyl-1-picrylhydrazyl), ABTS (2.2′-azino-Bis(3-ethylbenzothiazoline-6-sulphonic acid), TAC (total antioxidant capacity), FRAP (ferric-reducing antioxidant), reducing power assay, *β*-carotene bleaching inhibition, ferrous ion chelating, hydroxyl radical, hydrogen peroxide scavenging activity, and metal chelating activity. The antioxidant activities of *Ephedra* reported in the literature was illustrated in Table 2. Among these studies, Danciu et al. [90] showed that the Tunisian aerial parts of *E. alata* Decne, extracted with EtOH 70%, have an important antioxidant activity (CUPRAC) which is around 7453.18 µmol Trolox/g. Also, Benabderrahim et al. [23] found that the antioxidant contents, expressed by DPPH and ABTS, of EtOH/water (v/v) extracts of *E. alata* Decne were, respectively, 33.51 ± 0.05 mg TEAC/100 g and 37.86 ± 0.03 mg TEAC/100 g. Mighri et al. [45] showed that the chloroform fraction of Tunisian aerial parts of *E. alata* exhibited the highest antioxidant activity (TAC and DPPH) compared to methanol extract and butanol, ethyl acetate, and water fractions. The authors of this study reported that, compared with the methanol extract, butanol, aqueous, and chloroform fractions, ethyl acetate showed higher FRAP activity (21.36 mM TEq/g). From Algerian *E. alata* Decne ssp. *alenda*, Ziani et al. [100] found that the EtOH/water extract displayed the highest DPPH, reducing power, and *β*-carotene bleaching inhibition.

From northern Jordan, the *in vitro* antioxidant activities of the butanolic extract from the stem of *E alte* were assessed against DPPH, ABTS, and hydroxyl radicals [95]. In fact, butanolic extract showed different levels of radicals scavenging activity in a dose-dependent manner over the range of 5–500 *μ*g/mL concentration, indicating the high antioxidative capacity of the extract. The IC_50_ values of the extract were 66.4, 50.2, 43.5, and 77.1 *μ*g/mL for DPPH, ABTS, hydroxyl radicals, and the ferrous ion chelating activity, respectively. Hegazy et al. [96] evaluated the antioxidant activities of the five wild underutilized fruits in the mountains of southwest Saudi Arabia (*Coccinia grandis* (L.) Voigt, *Diospyros mespiliformis* Hochst. ex A. DC., *Cissus rotundifolia* (L.), *E. foeminea* Forssk., and *Grewia villosa* Willd.). Corresponding to this study, methanol extract of *E. foeminea* displayed antioxidant activity higher than 50% even at low concentration of 0.6 mg/mL. Shawarb et al. [113] and Jaradat et al. [114] found that the leaves of Palestinian *E. alata* showed a good antioxidant activity. This activity was evaluated by the free radical scavenging as 15.85 *μ*g/mL (IC_50_) and 75.02% at 100 *μ*g/mL, respectively. Kallassy et al. [97] evaluated the antioxidant capacity of various solvent (distilled water, ethanol, and methanol) extracts of Lebanese *E. campylopoda* stems. The different extracts showed varying antioxidant potential, and their DPPH scavenging capacities were in the following order: ethanolic extract (IC_50_ = 125 *μ*g/mL) >methanolic extract (IC_50_ = 150 *μ*g/mL) >aqueous extract (IC_50_ = 300 *μ*g/mL). On the other hand, this study showed that methanolic extract had the most efficient Fe^2+^ chelating capacity (IC_50_ = 1 mg/mL) in comparison to both the ethanolic and the aqueous extracts, which presented IC_50_ values of more than 1.5 mg/mL.

Khan et al. [115] reported that the ethyl acetate fraction of Pakistani *E. gerardiana* (root and stem) presented more significant free radical scavenging potential than the methanol extract, chloroform, n-hexane, and n-butanol fractions, and the mean values ranged, respectively, from 21.49 to 2.96 *μ*g/mL. It should be noted that the stem of *E. gerardiana* gave the maximum antioxidant yield and chloroform gave the lowest one (IC_50_ = 22.73 *μ*g/mL). Extracted by solvent mixture of ethanol/methanol/water of aerial parts of Pakistani *E. intermedia*, the antioxidant activity, tested by DPPH radical procedure, was 90.08 at 100 *μ*g/mL [103].

Mellado et al. [94] studied the antioxidant (DPPH, FRAP, and TRAP assays) activity of Chilean *E. chilensis* K. The DPPH assay showed that the hexanoic extract had a poor activity (*P* < 0.05) compared with the positive controls (trolox and gallic acid). Dichloromethane (CH_2_Cl_2_) and ethanolic extracts showed similar activities, and these activities are different from the activities of trolox and gallic acid (*P* < 0.05). For the FRAP assay, the CH_2_Cl_2_ and EtOH extracts show better antioxidant activity than the positive controls (*P* < 0.05). Concerning the TRAP assay, Hex extract was the least active of all of the tested extracts compared with the positive controls (gallic acid and BHT) with significant differences (*P* < 0.05).

### 5.2. Antimicrobial Activity

Antimicrobial efficacy of *Ephedra* species extracts has been described in several studies using in vitro methods such as agar disc diffusion assays and/or minimum inhibitory concentration (MIC). The *in vitro* antimicrobial activity against a number of pathogenic and drug-resistant bacteria and fungi is presented in Table 3. Using both in *vitro* agar diffusion and MIC (minimum inhibitory concentration) Danciu et al. [90] showed that the hydroalcoholic extract of *E. alata* Decne, collected from Djerba (Tunisia), had a bactericidal effect against *Staphylococcus aureus* ATCC 25923 and *Enterococcus faecalis* ATCC 51299 and fungicide impacts on *Candida albicans* ATCC 10231 and *Candida parapsilosis* ATCC 22019. Palici et al. [118] studied the antibacterial activity of ethanol/water extract of *E. alata* var. *alenda*, collected from Tunisian region of Sahara. This studied extract demonstrated a notable inhibition against methicillin-resistant *Staphylococcus aureus* (MRSA) ATCC 29213. Other authors demonstrated that the decoction and infusion of hydroethanolic extract of E. al*ata* exhibited a MIC value of 5 mg/mL against methicillin-susceptible *S. aureus* (MSSA) and methicillin-resistant *S. aureus* (MRSA) [100]. In this study, infusions and decoctions had a weak effect, except against *E. coli* strains, which was the most susceptible microorganism with a MIC value of 0.625 mg/mL. On the contrary, high antibacterial and antifungal effects of this plant were previously reported in extracts prepared with water, methanol, and acetonitrile, with the latter exhibiting the most potent effect against all the microorganisms supplied by the Regional Center for Mycology and Biotechnology (RCMB), Al-Azhar University, Cairo, Egypt [121].

Ethanolic and hydroalcoholic herb extract of Iranian *E. sinica* was assayed against standard and clinical *Pseudomonas aeruginosa*, and then the MIC and MBC (minimum bactericidal concentration) were assayed [119]. The results showed the lowest MIC values of ethanolic herb extract were, respectively, 25 and 12.5 *μ*g/mL, but the lowest MIC values of the hydroalcoholic herb extract were 25 and 25 *μ*g/mL, respectively. Equally, the lowest MBC values of ethanolic herb extract on clinical and standard strains of *P. aeruginosa* were 50 and 25 *μ*g/mL, respectively; however, the lowest MBC values were 25 and 25 *μ*g/mL, respectively. The biosynthesized *E. procera* nanoparticles (EpNPs) exhibited considerable activity against *E. coli* ATCC 25922 and *B. subtilis* ATCC 6633 with MICs of 11.12 *μ*g/mL and 11.33 *μ*g/mL, respectively. Nevertheless, EpNPs showed moderate activity against *P. aeruginosa* while the *S. epidermidis* and *S. aureus* strains were found resistant. Equally, EpNPs showed considerable antifungal activity against *A. flavus* and *A. Niger*, but moderate activity against *Mucor* spp [120]. These authors have proven that EpNPs showed antifungal activity against *A*. *flavus*, *A. Niger*, and *Mucor* spp. with a diameter of inhibitory zone equal to 14.2, 15.8, and 11 mm, respectively. Four extracts of Pakistani *E. vulgaris* (CHCl_3_, MeOH, EtOH, and water) were used against three pathogen bacteria, namely, *Streptococcus pneumonia*, *Pseudomonas aeruginosa*, and *Klebsiella pneumonia* [122]. It was noted from the results that chloroform and aqueous extracts have no inhibition effects against *P. aeruginosa.* The maximum inhibitory effects (17.16 mm inhibition zone) in chloroform extract against *S. pneumoniae* and minimum inhibition activities (8.70 mm zone of inhibition) in the extract of ethanol against *P. aeruginosa* were observed.

## 6. Biomedicine and Pharmacotherapy Activity 

Pharmacological activities of extracts from different species of the worldwide genus *Ephedra*, published in the period 2015–2019, are well documented in Table 4. The evaluation of the antiproliferative, proapoptotic, and cytotoxic potential against the MCF-7 breast cancer cell line of the ethyl acetate (EA) extract of the aerial part of *E. alata* Decne was reported by Danciu et al. [90]. In this study, the antiproliferative activity of EA started from a concentration of 10 *μ*g/mL, with a cell growth inhibition of 19.68%. For the highest tested concentration, 30 *μ*g/mL, the growth inhibition percentage was 56.45. The cytotoxicity assessment revealed that the EA manifested a significant difference in cytotoxic potential, displaying a cytotoxicity percentage above 13%. The potential antimigratory activity of the EA extract on MCF-7 human breast adenocarcinoma cells was verified by means of a wound-healing technique. In this regard, on the MCF-7 cells' migration EA had a strong inhibitory effect and showed a wound-healing rate below 5% after an interval of 24 h. The cytotoxicity assessment revealed that the EA extract manifested a significant difference in the cytotoxic potential when compared to the positive control (DMSO), displaying a cytotoxicity percentage of ∼13%. To investigate the apoptotic potential of EA at the selected concentration, MCF-7 cells were treated with 30 *μ*g/mL for 72 h, and the cells' nuclei were analyzed by DAPI (4′,6′-diamidino–2-phenylindole) staining. In this line, Danciu et al. [90] showed that the control cells exhibit a normal organization, with a large, round nucleus, a clear nucleolus, and uniform chromatin density. However, after treatment, the MCF-7 cells manifested morphological changes distinctive for apoptosis induction, such as chromatin condensation.

Jaradat et al. [114] investigated the use of herbal remedies by women living with breast cancer in the West Bank of Palestine by a questionnaire-based cross-sectional descriptive study; the questionnaire was distributed to 115 patients. This study revealed that *E. alata* was the most commonly used plant species in the treatment of breast cancer. Leaves and seeds of *E. alata* were the most commonly used parts, and decoction was the most commonly used method of preparation. Jordanian *E. aphylla* extracts (methanol, chloroform, ethyl acetate, n-hexane, and water) were tested to evaluate antiproliferative potential [122]. The authors observed that all extracts displayed strong antiproliferative potential against the tested cell lines (breast cancer cell lines (T47D, MCF-7) and Vero cell line (African green monkey kidney)). Moreover, *E. aphylla* extracts showed a little cytotoxicity activity against the Vero normal cell line. The antiproliferative activity of various solvent extracts against MFC7 cell line was in the order of aqueous > methanol > chloroform > ethyl acetate > n-hexane [122].

Kallassy et al. [97] studied the anti-inflammatory and antiproliferative potential of ethanol, methanol, and water extracts of Lebanese *E. campylopoda* stems. The anti-inflammatory capacity was estimated both by evaluating RAW 264.7 murine macrophage cells-mediated secretion of PGE_2_ using ELISA technique, and by quantifying the mRNA level of the proinflammatory cytokines (IL-*α*, IL-*β*, and IL-6), chemokines (CCL3 and CCL4), and inflammation-inducible COX-2 and iNOS enzymes using quantitative real-time PCR (qRT-PCR). By using the XTT viability assay, the antiproliferative potential of *E. campylopoda* was determined. This study confirmed that the alcoholic extracts showed more potent anti-inflammatory and antiproliferative capacities than aqueous extract [97]. Hoshyar et al. [123] examined the anticancer effects of *E. sarcocarpa* on proliferation of breast cancer, MCF-7, and epithelial normal MCF-10A cells. These authors evaluated the effect of *E. sarcocarpa* aqueous extracts on cell proliferation and investigated the cytotoxic effects at concentrations ranging between 0 and 3 mg/mL on the growth of human breast cancer (MCF-7) and normal mammalian (MCF10-A) cells after different time incubation (0–72 h) using MTT (the methylthiazolyldiphenyl-tetrazolium bromide) assay. This study uncovers that the treatment of MCF-7 cells with the *E. sarcocarpa* aqueous extract (0.25, 0.5, and 0.75 mg/mL) significantly decreased cell viability and increased cell death percentage by increasing extract concentration after 72 h. Parallel treatments of the normal cells with this extract indicted a much less inhibitory effect on the viability of MCF10-A cells.

Park et al. developed *E. sinica* Stapf. (ES) extract-capped gold nanoparticles (ES-GNs) and investigated their antineuroinflammatory properties in microglia [116]. For this purpose, antineuroinflammatory properties of ES-GNs on production of proinflammatory mediators (nitric oxide, prostaglandin *E*_2_, and reactive oxygen species) and cytokines (tumor necrosis factor-*α*, IL-1*β*, and IL-6) in lipopolysaccharide- (LPS-) stimulated microglia were well investigated by ELISA and flow cytometry. In this regard, ES-GNs significantly attenuated LPS-induced production of proinflammatory mediators and cytokines, which was related to suppressed transcription and translation of inducible nitric oxide synthase and cyclooxygenase-2, determined by RT-PCR and western blotting. These authors hypothesized that antineuroinflammatory properties of ES-GNs were mediated by AMP-activated protein kinase and nuclear erythroid 2-related factor 2/antioxidant response element signaling. In 2017, Lee et al. [130] evaluated the effects and molecular targets of methanolic extract of dried stems and leaves of *E. sinica* Stapf. and *E. intermedia* Schrenk (EHM) on high-fat diet- (HFD-) induced hyperlipidemic ICR mice. According to these authors, results showed that EHM administration for 3 weeks significantly (*P* < 0.05) decreased total cholesterol (TC) and triglyceride levels without altering body weight (BW) in mice, and gene expression levels in the livers of EHM-treated mice were restored at 34.0% and 48.4% of those up- or downregulated by hyperlipidaemia, respectively. Hyuga et al. [107] confirmed that ephedrine alkaloids-free *Ephedra* herb extract (EFE) suppressed hepatocyte growth factor- (HGF-) induced cancer cell motility by preventing both HGF-induced phosphorylation of c-Met and its tyrosine kinase activity. Equally, this study displayed the analgesic effect of EFE and the anti-influenza virus activity by showing inhibition of MDCK cell infection in a concentration-dependent manner. All assessments of toxicity, even after repeated oral administration, suggest that EFE would be a safer alternative to *Ephedra* herb.

Oshima et al. [108] established a preparation method for EFE : ephedrine alkaloids-free *Ephedra* herb extract (EH) and revealed its chemical composition, including the content of herbacetin, a flavonoid aglycon. In addition, these authors showed the antiproliferative effects of EFE against the H1975 non-small-cell lung cancer (NSCLC) cell line. It should be noted that the antiproliferative effect of EFE against H1975 cells was comparable to that of EH extract. The *Ephedra*-*Gypsum* extracts at test dose (6, 12, 24 g/kg) significantly and dose-dependently attenuated yeast-induced fever in rats. The *Ephedra*-*Gypsum* extracts also prolonged the latent period, reduced ovalbumin- (OVA-) induced increases in eosinophils and white blood cell (WBC), and decreased the wet and dry weight ratio of the lungs in the antiasthmatic test.

## *7. Ephedra* Toxicity

Although *Ephedra* metabolites are naturally occurring alkaloids that can be derived from evergreens worldwide and have been used as medicinals, recent studies reported that ephedrine has various adverse effects on organisms such as hepatitis, angle closure glaucoma, nephrolithiasis, neurodegenerative diseases, and cardiovascular toxicity. Few of these side effects are reversible whereas others are irreversible and may even lead to death [28]. Several recent reviews have documented the dangerous nature of using these “drugs” unsupervised, including multiple deaths, and the FDA is currently reviewing ephedrine's use in the alternative medicine industry. Powell et al. [131] reported the toxicity ephedrine nephrolithiasis in a patient using an energy supplement, Ma Huang extract, which contains ephedrine. Although previously not reported, The Louis C. Herring and Company kidney stone database shows that this is an endemic complication of ephedrine with hundreds of previous episodes.

Nauffal and Gabardi [132] found that the use of 40–3,000 mg/day of *E. sinica* for several months can cause nephrolithiasis with flank pain, hematuria, and renal dysfunction. On the other hand, recent studies have reported that *Ephedra* herb containing many pharmacologically active alkaloids, principally ephedrine, has been reported to cause acute hepatitis. In this context, Lee et al. [133] investigated hepatotoxicity and key regulation of mitophagy in ephedrine-treated LX-2 cells. However, mitochondrial swelling and autolysosome were observed in ephedrine-treated cells. Also, ephedrine inhibited mitochondrial biogenesis, and the mitochondrial copy number was decreased. Moreover, antioxidants can serve as therapeutic targets for ephedrine-induced hepatotoxicity [133]. Equally, it is important to note that *Ephedra* species have been implicated in causing liver injury in case reports [134]. Similarly, speculation has also indicated that the alkaloid ephedrine may be hepatotoxic, based upon case reports, and associated with liver injury [135]. An *in vitro* assay using human hepatoblastoma cells (HepG2) demonstrated that when *Ephedra* (Ma Huang) extracts were normalized for their ephedrine content, they displayed greater cytotoxicity relative to ephedrine itself, indicating that there may be other constituents responsible for toxicity [133]. While ephedrine and pseudoephedrine showed cytotoxicity in the HepG2 cell line, the concentrations required (i.e., >300 *μ*g/mL) again indicate that such data are likely irrelevant to in vivo administration.

The reported adverse reactions principally involve the cardiovascular system and are, in general, similar to other sympathomimetics. The most common side effect is hypertension with a risk of hemorrhagic stroke. Also ischemic stroke due to vasoconstriction and likely platelet aggregation can occur after its consumption [136]. Although the risk of hemorrhagic stroke with pseudoephedrine seems to be lower, it can occur and might result in death. The adverse reactions after *Ephedra* administration can more easily occur when it is used in combination with caffeine. This combination increases the effect of sympathomimetics, and the mechanisms will be discussed later [136].

Ephedrine is also found in dietary supplements that promote short-term weight loss, but those are now illegal in the USA. However, in traditional Chinese medicines that contain ephedrine, it is legal. The ephedrine quantity in dietary supplements taken orally is about 20 mg per serving, and doses are taken up to two to three times per day. It has been shown that labels of dietary supplements do not list ephedrine content or incorrectly report the amount of ephedrine in these products. Ephedrine has been associated with cardiovascular dysfunction such as myocardial infarction, severe hypertension, myocarditis, and lethal cardiac arrhythmias. The typical dose of ephedrine used for bronchodilation is 25–50 mg, but ephedrine doses associated with adverse events were less than this amount [137].

The use of drug-herb interaction causes irreversible neurodegenerative diseases. For example, a 29-year-old male has been injected with an intravenous mixture of pseudoephedrine (extracted from *Ephedra*), potassium permanganate, and acetylsalicylic acid two to three times a day for 9 years and a half. Throughout these years, the patient developed many symptoms including speech disturbance, inability to walk independently, postural instability, tremor, and dystonia. The patient was diagnosed with manganese toxicity which leads to irreversible neurodegenerative disorder due to the long exposure to *E. sinica* [28].

## 8. Conclusion

*Ephedra* natural products have attracted more and more attention since they can exhibit complementary biological and therapeutic effects against diseases. Historically, *Ephedra* may even have been diverse and widespread at that time and the corresponding fossils document that *Ephedra* was already present in the Early Cretaceous. Further testing and development of methods for molecular dating will be needed to clarify conflicts between molecular signals and the fossil record.

In this review, we summarized the chemical components isolated and identified by MS. Further instrument sophistication in coupling several systems such as multidimensional chromatography with NMR and MS in series is already occurring. The prediction of the future for promising approaches involves the application of HPLC with ESI time of flight mass spectrometers and ESI FT ion cyclotron resonance mass spectrometers. An increased emphasis on microcapillary columns with nanotechnology ESI systems driven partly by environmental issues seems inevitable.

Additionally, biopharmacological effects, such as anticancer, anti-inflammatory, antitumor, hepatoprotective, antioxidant, and antimicrobial activities, have been well discussed. The relationship between the *Ephedra* natural products structure and its pharmacological activity needs to be further studied. In this context, the mechanisms of action of phytochemical *Ephedra* content can provide guidance for its clinical application.

## Figures and Tables

**Table 1:** Selected high-resolution MS applications in the characterization of *Ephedra* species compounds (published in the period 2015–2019).

Source	Part	Solvent	Analyte	Mobile phase and gradient program	Analytical method	Detection (nm)	Reference
Tunisia	Aerial parts of *E. alata* Decne	70% EtOH	Gallic acid, protocatechuic acid, caffeic acid, epicatechin, p-coumaric acid, ferulic acid, rutin, rosmarinic acid, resveratrol, quercetin and kaempferol	A: water acidified with formic acid at pH 3; B: acetonitrile acidified with formic acid at pH 3 : 0.01–20 min, 5% B; 20.01–50 min, 5–40% B; 50–55 min, 40–95% B; and 55–60 min 95% B	LC-MS	280–320	[90]
	Aerial parts of *E. alata*	EtOH/water (50 : 50 v/v)	Quinic acid, gallic acid, 4-O-caffeoylquinic acid, syringic acid, p-coumaric acid, trans-ferulic acid, catechin, epicatechin, rutin, quercitrin (quercetin-3-O-rhamnoside), apigenin-7-O-glucoside, kaempferol, naringenin, luteolin, cirsilineol	The mobile phase was composed of A (0.1% formic acid in H_2_O, v/v) and B (0.1% formic acid in methanol, v/v): linear gradient elution: 0–45 min, 10–100% B; 45–55 min, 100% B	LC-ESI/MS/MSn	280	[23]
	Aerial parts of *E. alata*	70% MeOH then fractionation with hexane, DCM, EAc BuOH, and water	Quinic acid (1), gallic acid (2), protocatechuic acid (3), chlorogenic acid (3-O-caffeoylquinic acid) (4), caffeic acid (5), syringic acid (6), p-coumaric acid (7), trans-ferulic acid (8), o-coumaric acid (9), transcinnamic acid (10), 4-O-caffeoylquinic acid (11), 1,3-di-O-caffeoylquinic acid (12), 3,4-di-O-caffeoylquinic acid (13), 4,5-di-O-caffeoylquinic acid (14), rosmarinic acid (15), salvianolic acid (16), (+)-catechin (17), (−)-epicatechin (18), acacetin (19), apigetrin (apigenin-7-O-glucoside) (20), apigenin (21), quercitrin (quercetin-3-o rhamnoside) (22), kaempferol (23), cirsilineol (24), cirsiliol (25), hyperoside (quercetin-3-O-galactoside) (26), cynaroside (luteolin-7-O-glucoside) (27), luteolin (28), Naringenin (29), naringin (naringenin-7-O-rutinoside) (30), quercitrin (quercetin-3-O-rhamnoside) (31), rutin (quercetin-7-O-rutinoside) (32), and silymarin (33)	The mobile phase: A (0.2% acetic acid in 95% water and 5% MeOH) and B (0.2% acetic acid in 50% water and 50% acetonitrile) with a linear gradient elution: 0–45 min, 10–20% B; 45–85 min, 20–55% B; 85–97 min, 55–100% B; 97–110 min, 100% B; the initial conditions were held for 10 min as a re-equilibration step	HPLC-PDA-ESI/MS	(1) 240; (2) 272–218; (3) 259–294-220; (4)230–280; (5) 327–245-295; (6) 327–295-245; (7) 324–295-220; (8) 274–220; (9) 322–302–245–218; (10) 230–279; (11) 309–229-298; (12) 322–240-295; (13) 255–356; (14) 355–256; (15) 275–325-230; (16) 347–253-267; (17) 329–295–245–221; (18) 329–290-245; (19) 329–290-245; (20) 212–226–282–328; (21) 336–67; (22) 325–295–245–221; (23) 287–254–309–228; (24) 275–222-215; (25) 275–365; (26) 254–290- 366; (27) 228–288-332; (28) 337–267-225; (29) 287–231; (30) 347–253-266; (31) 344–273-225; (32) 343–247-225; and (33) 331–268	[45]
Algeria	Whole plant *E. alata* Decne ssp. *alenda*	- Infusion - Decoction - 80% EtOH	10 phenolic compounds: 2 myricetin-C-hexoside isomers (1 and 2); biochanin A 7-O-glucoside (Sissotrin) (3); 2 hydroxydaidzein-8-C-glucoside isomers (4 and 5); 5,5′-dihydroxy-methoxy-isoflavone-O-glucoside (6); hydroxydaidzein-8-C-glucoside isomer (7); quercetin-3-O-rutinoside (8); isorhamnetin-3-O-glucoside (9); and kaempferol-O-di-deoxyhexoside (10)	(A) 0.1% formic acid in water, and (B) acetonitrile: 15% B (0–5 min), 15%–20% B (5–10 min), 20–25% B (10–20 min), 25–35% B (20–30 min), and 35–50% B (30–40 min)	LC-DAD-ESI/MSn	(1) 291, 340; (2) 290, 340 (3) 255, 320; (4) 262,340 (5) 262,340 ; (6) 263,336 (7) 262,340 ; (8) 351 (9) 368; and (10) 263,348	[100]
Austria	Aerial parts of *E. sinica*	PET, DCM, EtOAc n-BuOH, EtOH, MeOH, or water	Epigallocatechin-4*β*-benzylthioether, epigallocatechin-4-benzylthioether stereoisomers, and epicatechin-4*β*-benzylthioether	*A* = water, *B* = acetonitrile. 0 min: 35% B, 20 min: 35% B, 30 min: 45% B, 40 min: 45% B, 45 min: 98% B60 min: S top; post- time 15 min	HPLC and HPLC-MS	254	[101]
Austria/Germany	Aerial parts of 8 *Ephedra* spp.	HCl (6.2%, v/v)	Ephedrine and pseudoephedrine	Acetonitrile, tetrahydrofuran, and water (38 : 5:57, v/v/v)	UPLC-UV	208	[102]
Palestine	Aerial parts of *E. alata*	EtOH, EtOH (80%), and water	Luteolin-7-O-glucuronide, myricetin-3-rhamnoside	The start was a 100% (*A*) that descended to 70% (*A*) in 40 minutes, then to 40% (*A*) in 20 minutes, and finally to 10% (*A*) in 2 minutes and stayed there for 6 minutes and then back to the initial conditions in 2 minutes	HPLC/PDA and HPLC/MS	350	[41]
Iran	Green stems from *E. major*	MeOH (80%)	Ephedrine	A mixture of 0/1% phosphoric acid (pH 4), 25 mM SDS, and 40% acetonitrile (10 : 1 v/v)	HPLC	210	[42]
Pakistan	Aerial plant of *E. intermedia*	70% EtOH and MeOH 70%	Ephedrine and pseudoephedrine	Buffer solution of H_3_PO_4_ at 0.25 M (pH 5.3), methanol, and acetonitrile in ratio 1 : 1: 8	HPLC	210	[103]
Korea	Aerial parts of *E. intermedia*	30% EtOH	Ephedrine and pseudoephedrine	60% solvent A (0–25 min), 60–40% solvent A (25–35 min), 40% solvent A (35–40 min), 40–20% solvent A (40–50 min), and 20% solvent A (50–60 min)	HPLC-UVD	210 and 254	[104]
	*E. sinica*	Distilled water for 22 h at 95 °C	Ephedrine (1), pseudoephedrine (2), rhein (3), aloe-emodin (4), emodin (5), chrysophanol (6), and physcion (7)	-For (1) and (2) the mixtures of HPLC-grade H_2_O buffered with 25 mM sodium dodecyl sulfate (solvent A) and acetonitrile (AcCN, solvent B) -For (3), (4), (5), (6), and (7) the mixtures of H2O, AcCN, and phosphoric acid (850 : 150 : 1) for 20 min - For (1) and (2): 60% solvent A for 40 min	HPLC	(1) 215; (2) 215 (3) 254; (4) 254 (5) 254; (6) 254, and (7) 254	[105]
	Stems of *E. intermedia*	70% EtOH	Ephedrine, pseudoephedrine, N-methylephedrine, N-methylpseudoephedrine, norephedrine, and norpseudoephedrine	Isocratic gradient 25 mM SDS in water (A) and acetonitrile (B)	HPLC-UV	215	[106]
Japan	*E. sinica*	Water at 95°C	Syringin; kaempferol 3-O-rhamnoside 7-O-glucoside; isovitexin 2″-O-rhamnoside; cinnamic acid; 6-hydroxykynurenic acid; 6-methoxykynurenic acid	0.1% formic acid in water (*A*) and 0.1% formic acid in methanol (B): 5% B (0–10 min), 5–75% B (10–70 min), 75–100% B (70–80 min), 100% B (80–90 min)	LC-PDA	210	[107]
	*E. sinica*	Hot water at 95 °C for 1 h	Vicenin-2 and isovitexin 2″-O-rhamnoside	0.1% formic acid (HCOOH) in water (*A*) − 0.1% HCOOH in MeOH (B) 5% B (0 min) ⟶ 50% B (40 min) ⟶ 100% B (50 min) ⟶ 100% B (55 min) ⟶ 5% B (55.1 min) ⟶ 5% B (60 min)	LC/Orbitrap MS	200–400	[108]
Taiwan	Aerial parts of *Ephedra*	Boiling	Ephedrine, amygdalin, glycyrrhizic acid, and carvedilol	5 mM NH_4_CH_3_CO_2_ (0.1% formic acid) as the aqueous phase (A) and 100% methanol (0.1% formic acid) as the organic phase (B); 20–70% B at 0–1 min, 70–90% B at 1–4 min, 90% B at 4–9 min, 90–20% B 9–10 min, 20% B at 10–13 min	UHPLC–MS/MS		[109]
China	Aerial parts of *Ephedra*	Water	Norephedrine, norpseudoephedrine, ephedrine, pseudoephedrine, and methylephedrine	A mixture of KH_2_PO_4_ (20 mmol/L)-acetonitrile (96 : 4, v/v)	HPLC	210	[110]
	*Ephedra* herb	ACN-ammonium acetate	Methylephedrine, ephedrine, and pseudoephedrine	Acetonitrile-ammonium acetate (pH 5.0; 0.195 M) (95 : 5, v/v)	HPLC	208	[111]
	Stems of *E. sinica*	EtOH, EtOAc, and BuOH	(S)–N-((1R,2S)-1-hydroxy-1-phenylpropan-2-yl)-5-oxopyrrolidine-2-carboxamide (1) and (3R)-3-O-*β*-D-glucopyranosyl-3-phenylpropanoic acid (2)	∗CH3OH/H2O (23%, v/v) (1) ∗ 25% MeOH in H2O, containing 0.1% formic acid (2)	LC/MSD	280	[112]

**Table 2:** Antioxidant activity of *Ephedra* species (published in the period 2015–2019).

Source	Part	Extraction	Method		Activity	References
Tunisia	Aerial parts of *E. alata* Decne	EtOH 70%	CUPRAC		7453.18 ± 2.5 µmol trolox/g	[90]
	Aerial parts of *E. alata*	EtOH/water (v/v)	DPPH		33.51 ± 0.05 mg TEAC/100 g	[23]
			ABTS		37.86 ± 0.03 mg TEAC/100 g	
	Aerial parts of *E. alata*	MeOH 70% (I); CHCL_3_ (II)EtOAc (III); BuOH (IV); and water (V)	TAC	(I)	125.50 ± 3.50 mg aa eq/g	[45]
				(II)	221.71 ± 8.90 mg aa eq/g	
				(III)	145.71 ± 13.1 mg aa eq/g	
				(IV)	130.29 ± 2.60 mg aa eq/g	
				(V)	56.29 ± 4.50 mg aa eq/g	
			DPPH	(I)	0.330 ± 0.004 mg/mL	
				(II)	0.454 ± 0.008 mg/mL	
				(III)	0.180 ± 0.002 mg/mL	
				(IV)	0.176 ± 0.002 mg/mL	
				(V)	-	
			FRAP	(I)	10.38 ± 0.04 mM TEq/g	
				(II)	18.32 ± 0.07 mM TEq/g	
				(III)	21.36 ± 0.04 mM TEq/g	
				(IV)	4.14 ± 0.03 mM TEq/g	
				(V)	0.82 ± 0.02 mM TEq/g	
Algeria	Whole plant of *E. alata*Decne ssp. *alenda*	Water (boiling)	DPPH (EC50)		450 ± 7 *μ*g/mL	[100]
			Reducing power (EC50)		108 ± 1 *μ*g/mL	
			*β*-carotene bleaching inhibition		131 ± 1 *μ*g/mL	
		Water (decoction)	DPPH (EC50)		455 ± 6 *μ*g/mL	
			Reducing power (EC50)		109 ± 3 *μ*g/mL	
			*β*-Carotene bleaching inhibition (EC50)		173 ± 3 *μ*g/mL	
		EtOH/water	DPPH (EC50)		540 ± 3 *μ*g/mL	
			Reducing power (EC50)		377 ± 4 *μ*g/mL	
			*β*-carotene bleaching inhibition (EC50)		502 ± 8 *μ*g/mL	
Jordan	Stems of *E*. *alata*	Petroleum ether and MeOH	DPPH (IC50)		66.4 ± 0.55 *μ*g/mL	[95]
			ABTS (IC50)		50.2 ± 1.2 *μ*g/mL	
			Ferrous ion (Fe2+) chelating		77.1 ± 1.1 *μ*g/mL	
			(IC50)			
			Hydroxyl radical (IC50)		43.5 ± 1.14 *μ*g/mL	
Saudi Arabia	Ripe fruits of *E. foeminea*	MeOH	DPPH (1 mg/mL)		68%	[96]
			Total antioxidant activity		60%	
			Hydrogen peroxide scavenging activity (1 mg/mL)		68%	
Palestine	Leaves of *E. alata*	MeOH	DPPH (IC50)		15.85 *μ*g/mL	[113]
	Aerial parts of *E. alata*	MeOH	DPPH (100 *μ*g/mL)		75.02 ± 1.67%	[114]
Lebanon	Fresh stems of *E. campylopoda*	Water	DPPH(IC50)		300 ± 4.4 *μ*g/mL	[97]
			Metal chelating activity (IC50)		>1.5 mg/mL	
		EtOH	DPPH (IC50)		125 ± 4.4 *μ*g/mL	
			Metal chelating activity (IC50)		>1.5 mg/mL	
		MeOH	DPPH (IC50)		150 ± 5.1 *μ*g/mL	
			Metal chelating activity (IC50)		1 ± 1.2 mg/mL	
Pakistan	Root and stem of *E. gerardiana*	MeOH	DPPH (root) (IC50)		14.94 ± 3.54 *μ*g/mL	[115]
		Water	DPPH (stem) (IC50)		3.44 ± 0.69 *μ*g/mL	
		n-Hx	DPPH (roots) (IC50)		21.49 ± 6.26 *μ*g/mL	
			DPPH (stem) (IC50)		13.92 ± 6.04 *μ*g/mL	
		CHCl_3_	DPPH (roots) (IC50)		6.38 ± 1.59 *μ*g/mL	
			DPPH (stem) (IC50)		22.73 ± 6.92 *μ*g/mL	
		EtOAc	DPPH (roots) (IC50)		2.96 ± 0.39 *μ*g/mL	
			DPPH (stem) (IC50)		2.73 ± 0.84 *μ*g/mL	
		n-BuOH	DPPH (roots) (IC50)		13.74 ± 2.71 *μ*g/mL	
			DPPH (stem) (IC50)		2.69 ± 0.26 *μ*g/mL	
	Aerial parts of *Ephedra*	EtOH/MeOH/water	DPPH (100 *μ*g/mL)		90.08 ± 1.37%	[103]
Korea	Stem of *E. sinica*	EtOH	DPPH (1 mg/mL)		75%	[116]
			ABTS (1 mg/mL)		80%	
Chile	Aerial parts of *Ephedra chilensis*	n-Hx	DPPH (IC50)		13.77 ± 0.37 mg/mL	[94]
			FRAP		3.90 ± 0.20TEAC mM	
			TRAP		0.28 ± 0.05TEAC mM	
		CH_2_Cl_2_	DPPH (IC50)		3.02 ± 0.02 mg/mL	
			FRAP		21.05 ± 0.18TEAC mM	
			TRAP		1.40 ± 0.07TEAC mM	
		EtOH	DPPH (IC50)		0.68 ± 0.01 mg/mL	
			FRAP		24.00 ± 0.43TEAC mM	
			TRAP		1.53 ± 0.06TEAC mM)	
	Leaves and stems of *E. chilensis*	EtOH	DPPH (1 mg/mL)		82%	[117]

**Table 3:** Antimicrobial activity extract from different species of the worldwide genus of *Ephedra*.

Source	Part	Extraction	Target microorganism	Activity		References
				MIC	IZ (mm)	
Tunisia	Aerial parts of *E. alata* Decne	EtOH 70%	*Klebsiella pneumonia* ATCC 700603	200 *μ*g/mL	7	[90]
			*Shigella flexneri* ATCC 12022	200 *μ*g/mL	7	
			*Salmonella enterica* ATCC 14028	200 *μ*g/mL	7	
			*Escherichia coli* ATCC 25922	200 *μ*g/mL	7	
			*Pseudomonas aeruginosa* ATCC 27853	200 *μ*g/mL	7	
			*Staphylococcus aureus* ATCC 25923	50 *μ*g/mL	9	
			*Enterococcus faecalis* ATCC 51299	100 *μ*g/mL	7	
			*Candida albicans* ATCC 10231	50 *μ*g/mL	10	
			*Candida parapsilosis* ATCC 22019	50 *μ*g/mL	10	
	Aerial parts of *E. alata* var. *alenda*	EtOH–water (1 : 1) -		50 mg/mL	9.5 mm	[118]
	*Bacillus subtilis* ATCC 6633					
			*Moraxella catarrhalis* ATCC 25238	-	7.5 mm	
			Methicillin-resistant *Staphylococcus aureus* ATCC 43300	>5	14.5 mm	
			*Staphylococcus aureus* ATCC 29213	-	9.5 mm	
Algeria	Whole plant of *E. alata* Decne ssp. *alenda*	Water (infusion	*Escherichia coli ESBL*	20 mg/mL		[100]
			*Escherichia coli*	20 mg/mL		
			*Klebsiella pneumoniae ESBL*	20 mg/mL		
			*Klebsiella pneumoniae*	20 mg/mL		
			*Morganella morganii*	20 mg/mL		
			*Pseudomonas aeruginosa*	>20 mg/mL		
			*Enterococcus faecalis*	20 mg/mL		
			*Listeria monocytogenes*	20 mg/mL		
			Methicillin-resistant *S. aureus*	10 mg/mL		
			Methicillin-susceptible *Staphylococcus aureus*	10 mg/mL		
		Water (decoction)	*Escherichia coli ESBL*	20 mg/mL		
			*Escherichia coli*	20 mg/mL		
			*Klebsiella pneumoniae ESBL*	20 mg/mL		
			*Klebsiella pneumoniae*	20 mg/mL		
			*Morganella morganii*	>20 mg/mL		
			*Pseudomonas aeruginosa*	>20 mg/mL		
			*Enterococcus faecalis*	20 mg/mL		
			*Listeria monocytogenes*	20 mg/mL		
			Methicillin-resistant *S. aureus*	20 mg/mL		
			Methicillin-susceptible *Staphylococcus aureus*	20 mg/mL		
		EtOH/H2O	*Escherichia coli ESBL*	5 mg/mL		
			*Escherichia coli*	5 mg/mL		
			*Klebsiella pneumoniae ESBL*	10 mg/mL		
			*Klebsiella pneumoniae*	10 mg/mL		
			*Morganella morganii*	20 mg/mL		
			*Pseudomonas aeruginosa*	20 mg/mL		
			*Enterococcus faecalis*	10 mg/mL		
			*Listeria monocytogenes*	10 mg/mL		
			Methicillin-resistant *S. aureus*	5 mg/mL		
			Methicillin-susceptible *Staphylococcus aureus*	5 mg/mL		
Iran	*E. sinica*	EtOH	*Pseudomonas aeruginosa* ATCC 27853	12.5 *μ*g/mL		[119]
Pakistan	Aerial parts of *E. procera*	H_2_O	*B*. *subtilis* ATCC 6633	11.33 *μ*g/mL	15.2	[93]
		(5 *μ*L (100 *μ*g/disc))	*P*. *aeruginosa* ATCC 9721	100 *μ*g/mL	11	
			*E. coli* ATCC 25922	11.12 *μ*g/mL	19.2	
			*S. epidermidis* ATCC 12228	-	-	
			*K. pneumoniae* ATCC 1705)	33.3 *μ*g/mL	14.2	
			*S. aureus* ATCC 6538	-	-	
			*A. fumigatus* FCBP 66		13	
			*A. flavus* FCBP 0064		14.2	
			*A. Niger* FCBP 0198		15.8	
			*Mucor* spp. FCBP 0300		11	
Pakistan	Dry stems of *E. vulgaris*	MeOH	*S. pneumoniae*		15.36	[120]
			*Pseudomonas aeruginosa*		10.36	
			*Klebsiella pneumoniae*		12.70	
		EtOH	*S. pneumoniae*		15.30	
			*Pseudomonas aeruginosa*		8.70	
			*Klebsiella pneumoniae*		11.60	
		CHCl_3_	*S. pneumoniae*		17.16	
			*Pseudomonas aeruginosa*		0	
			*Klebsiella pneumoniae*		12.63	
		Water	*S. pneumoniae*		13.26	
			*P. aeruginosa*		0	
			*K. pneumoniae*		13.70	

**Table 4:** Pharmacological activity of extracts from different species of the worldwide genus *Ephedra* (published in the period 2015–2019).

Source	Part	Extract	Therapy	Model	References
Tunisia	Aerial part of *E. alata*	Ethyl acetate	Antiproliferative, proapoptotic, and cytotoxic potential	MCF-7 human breast cancer cells	[90]
Palestine	Aerial parts of *E. alata*	Decoction	Anticancer	115 breast cancer patients	[114]
Jordan	Aerial parts of *E. aphylla*	MeOH, methanol, CHCl_3_, EtOAc, n-Hx, and water	Anti-inflammatory	The inhibition of albumin denaturation assay	[122]
			Antiproliferative	Breast cancer cell lines (T47D, MCF-7) and Vero cell line (African green monkey kidney)	
Lebanon	Stems of *E. campylopoda*	EtOH, MeOH and water	Anti-inflammatory	RAW 264.7, a murine monocyte/macrophage cell line	[97]
			Antiproliferative	Human leukemic T cell line	
Iran	Stems and leaves of *E. sarcocarpa*	Water	Anticancer	Human breast adenocarcinoma (MCF-7) and human normal breast epithelial (MCF10A) cell lines	[123]
	Aerial parts of *Ephedra*	Water	Antidiabetic and antihyperlipidemic	40 male BALB/cArc Wistar rats aged eight to ten weeks (200 to 250 g)	[124]
Pakistan	Aerial parts of *E.* g*erardiana*	EtOH 70%, EtOAc, n-BuOH, and water	Antiarthritic	-Young and healthy male and female Sprague-Dawley rats -Human red blood cell (HRBC) -Egg albumin -Protein (BSA)	[125]
Korea	Stem of *E. sinica*	Water	Antineuroinflammatory	Mouse primary microglia and immortal BV-2 mouse microglial cells	[116]
	Dried stems and leaves of *E. sinica* Stapf., *E*. *intermedia Schrenk, E. equisetina*	MeOH	Antihyperlipidemic	6-week-old male ICR mice weighing 20 to 25 g	[126]
Japan	*Ephedra*	Water	Analgesic	Specific pathogen-free ddY mice (5 weeks old, male)	[127]
	*E. sinica*	Water	Anti-influenza	Madin–Darby canine kidney (MDCK) cells	[107]
			Anticancer	MDA-MB-231 human breast cancer cells	
			Analgesic	ICR male mice (5 weeks of age, 8 mice per group)	
	*E. sinica*	Water	Antiproliferative	H1975 non-small-cell lung cancer (NSCLC) cell line	[128]
Taiwan	*Ephedra*	Water	Antipyretic	Male Sprague-Dawley rats (200–250 g)	[129]
			Antitussive	The eligible Guinea pigs	
China	*Ephedra*	Water	Antipyretic and antiasthmatic	Male Wistar rats (6 weeks old, 160–200 g, license number: SCXK 2011–0015), male SD rats (5 weeks old, 100–150 g, license number: SCXK 2011– 0015)	[110]
Chile	Aerial parts of *E. chilensis*	Hexane, dichloromethane and EtOH	Antiproliferative	MCF-7 (human breast cancer), HT-29 (human colon cancer), PC-3 and DU-145 (human prostate cancer), and CoN (human colon epithelial cells CCD 841)	[94]

## References

[1] Anisuzzman M., Hasan M. M., Acharzo A. K., Das A. K., Rahman S. (2017). *In vivo* and *in vitro* evaluation of pharmacological potentials of secondary bioactive metabolites of *Dalbergia candenatensis* leaves. *Evidence-Based Complementary and Alternative Medicine*.

[2] Sarfraz I., Rasul A., Jabeen F. (2017). *Fraxinus*: a plant with versatile pharmacological and biological activities. *Evidence-Based Complementary and Alternative Medicine*.

[3] Aktar K., Foyzun T. (2017). Phytochemistry and pharmacological studies ofCitrus macroptera: a medicinal plant review. *Evidence-Based Complementary and Alternative Medicine*.

[4] Sbhatu D. B., Abraha H. B. (2020). Preliminary antimicrobial profile of *Solanum incanum* L.: a common medicinal plant. *Evidence-Based Complementary and Alternative Medicine*.

[5] Biswas N. N., Acharzo A. K., Anamika S., Khushi S., Bokshi B. (2017). Screening of natural bioactive metabolites and investigation of antioxidant, antimicrobial, antihyperglycemic, neuropharmacological, and cytotoxicity potentials ofLitsea polyanthaJuss. Ethanolic root extract. *Evidence-Based Complementary and Alternative Medicine*.

[6] Blowman K., Magalhães M., Lemos M. F. L., Cabral C., Pires I. M. (2018). Anticancer properties of essential oils and other natural products. *Evidence-Based Complementary and Alternative Medicine*.

[7] Agyare C., Akindele A. J., Steenkamp V. (2019). Natural products and/or isolated compounds on wound healing. *Evidence-Based Complementary and Alternative Medicine*.

[8] Arena A. C., Kassuya C. A. L., Fernandes G. S. A., Scarano W. R. (2019). Toxic versus therapeutic effects of natural products on reproductive disorders. *Evidence-Based Complementary and Alternative Medicine*.

[9] Karim N., Abdelhalim H., Gavande N., Khan I., Khan H. (2018). Natural products as an emerging therapeutic alternative in the treatment of neurological disorders. *Evidence-Based Complementary and Alternative Medicine*.

[10] Cabral C., Efferth T., Pires I. M., Severino P., Lemos M. F. L. (2018). Natural products as a source for new leads in cancer research and treatment. *Evidence-Based Complementary and Alternative Medicine*.

[11] Wang X., Mao M., Liu S., Xu S., Yang J. (2019). A comparative study of bolus norepinephrine, phenylephrine, and ephedrine for the treatment of maternal hypotension in parturients with preeclampsia during cesarean delivery under spinal anesthesia. *Medical Science Monitor*.

[12] Di S., Wang Y., Han L. (2019). The intervention effect of traditional Chinese medicine on the intestinal flora and its metabolites in glycolipid metabolic disorders. *Evidence-Based Complementary and Alternative Medicine*.

[13] Alsayari A., Almghaslah D., Khaled A. (2018). Community pharmacists’ knowledge, attitudes, and practice of herbal medicines in asir region, kingdom of Saudi Arabia. *Evidence-Based Complementary and Alternative Medicine*.

[14] Tilahun M., Etifu M., Shewage T. (2019). Plant diversity and ethnoveterinary practices of Ethiopia: a systematic review. *Evidence-Based Complementary and Alternative Medicine*.

[15] Ninh S. (2017). A review on the medicinal plant *Dalbergia odorifera* species: phytochemistry and biological activity. *Evidence-Based Complementary and Alternative Medicine*.

[16] Ali S. A., Sharief N. H., Mohamed Y. S. (2019). Hepatoprotective activity of some medicinal plants in Sudan. *Evidence-Based Complementary and Alternative Medicine*.

[17] Lu C.-l., Li X.-f. (2019). A review ofOenanthe javanica(blume) DC. As traditional medicinal plant and its therapeutic potential. *Evidence-Based Complementary and Alternative Medicine*.

[18] Kim K., Park K.-I. (2019). A review of antiplatelet activity of traditional medicinal herbs on integrative medicine studies. *Evidence-Based Complementary and Alternative Medicine*.

[19] Andrade-Cetto A., Cruz E. C., Cabello-Hernández C. A., Cárdenas-Vázquez R. (2019). Hypoglycemic activity of medicinal plants used among the Cakchiquels in Guatemala for the treatment of type 2 diabetes. *Evidence-Based Complementary and Alternative Medicine*.

[20] Zhou Y.-X., Zhang R.-Q., Rahman K., Cao Z.-X., Zhang H., Peng C. (2019). Diverse pharmacological activities and potential medicinal benefits of geniposide. *Evidence-Based Complementary and Alternative Medicine*.

[21] Tsioutsiou E. E., Giordani P., Hanlidou E., Biagi M., De Feo V., Cornara L. (2019). Ethnobotanical study of medicinal plants used in central Macedonia, Greece. *Evidence-Based Complementary and Alternative Medicine*.

[22] Iqbal A., Khera R. A., Hanif M. A., Ayub M. A., Zafar M. N. (2020). Ma-Huang. *Medicinal Plants of South Asia*.

[23] Benabderrahim M. A., Yahia Y., Bettaieb I., Elfalleh W., Nagaz K. (2019). Antioxidant activity and phenolic profile of a collection of medicinal plants from Tunisian arid and Saharan regions. *Industrial Crops and Products*.

[24] Wang Q., Yang Y., Zhao X. (2006). Chemical variation in the essential oil ofEphedra sinica from Northeastern China. *Food Chemistry*.

[25] Schaneberg B. T., Crockett S., Bedir E., Khan I. A. (2003). The role of chemical fingerprinting: application to *Ephedra*. *Phytochemistry*.

[26] Caveney S., Charlet D. A., Freitag H., Maier-Stolte M., Starratt A. N. (2001). New observations on the secondary chemistry of world *Ephedra* (Ephedraceae). *American Journal of Botany*.

[27] Yang Y., Lin L., Ferguson D. K., Wang Y. (2018). Macrofossil evidence unveiling evolution of male cones in Ephedraceae (Gnetidae). *BMC Evolutionary Biology*.

[28] Al Dhamen M., Ahmad R., Ahmad N., Naqvi A. A. (2019). Clinical uses and toxicity of Ephedra sinica: an evidence-based comprehensive retrospective review (2004-2017). *Pharmacognosy Journal*.

[29] Eng Y. S., Lee C. H., Lee W. C., Huang C. C., Chang J. S. (2019). Unraveling the molecular mechanism of traditional Chinese medicine: formulas against acute airway viral infections as examples. *Molecules*.

[30] Sõukand R., Pieroni A., Biró M. (2015). An ethnobotanical perspective on traditional fermented plant foods and beverages in Eastern Europe. *Journal of Ethnopharmacology*.

[31] Hsu D.-Z., Liu C.-T., Chu P.-Y., Li Y.-H., Periasamy S., Liu M.-Y. (2013). Sesame oil attenuates ovalbumin-induced pulmonary edema and bronchial neutrophilic inflammation in mice. *BioMed Research International*.

[32] Thakur A., Pathak S. R. (2018). Introduction to medicinally important constituent from Chinese medicinal plants. *In Synthesis of Medicinal Agents from Plants*.

[33] Lee M. (2011). The history of *Ephedra* (ma-huang). *Journal of the Royal College of Physicians of Edinburgh*.

[34] Al-Salihi B. (2016). Ma huang (*ephedrae herba*): setting the record straight. *Journal of Chinese Medicine*.

[35] White L. B., Foster S. (2003). *The Herbal Drugstore: The Best Natural Alternatives to Over-the-counter and Prescription Medicines!*.

[36] Lipka A. F., Vrinten C., van Zwet E. W. (2017). Ephedrine treatment for autoimmune Myasthenia gravis. *Neuromuscular Disorders*.

[37] Andraws R., Chawla P., Brown D. L. (2005). Cardiovascular effects of *Ephedra* alkaloids: a comprehensive review. *Progress in Cardiovascular Diseases*.

[38] Cruz A., Padilla-Martínez I. I., Bautista-Ramirez M. E. (2018). Ephedrines as chiral auxiliaries in enantioselective alkylation reactions of acyl ephedrine amides and esters: a Review. *Current Organic Synthesis*.

[39] Cruz A., Esther Bautista Ramirez M. (2011). Ephedrines and their acyclic derivatives. *Current Organic Synthesis*.

[40] Rayan M., Abu-Farich B., Basha W., Rayan A., Abu-Lafi S. (2020). Correlation between antibacterial activity and free-radical scavenging: *in-Vitro* evaluation of polar/non-polar extracts from 25 plants. *Processes*.

[41] Al-Rimawi F., Abu-Lafi S., Abbadi J., Alamarneh A. A. A., Sawahreh R. A., Odeh I. (2017). Analysis of phenolic and flavonoids of wild *Ephedra alata* plant extracts by LC/PDA and LC/MS and their antioxidant activity. *African Journal of Traditional, Complementary and Alternative Medicines*.

[42] Aghdasi M., Mofid Bojnoordi M., Mianabadi M., Nadaf M. (2015). Chemical components of theEphedra majorfrom Iran. *Natural Product Research*.

[43] Dehkordi N. V., Kachouie M. A., Pirbalouti A. G., Malekpoor F., Rabei M. (2015). Total phenolic content, antioxidant and antibacterial activities of the extract of *Ephedra procera* fisch. et mey. *Acta Poloniae Pharmaceutica Drug Research*.

[44] Parsaeimehr A., Sargsyan E., Javidnia K. (2010). A comparative study of the antibacterial, antifungal and antioxidant activity and total content of phenolic compounds of cell cultures and wild plants of three endemic species of *Ephedra*. *Molecules*.

[45] Mighri H., Akrout A., Bennour N., Eljeni H., Zammouri T., Neffati M. (2019). LC/MS method development for the determination of the phenolic compounds of Tunisian *Ephedra alata* hydro-methanolic extract and its fractions and evaluation of their antioxidant activities. *South African Journal of Botany*.

[46] Roy M., Datta A. (2019). Fundamentals of phytochemicals. *Cancer Genetics and Therapeutics*.

[47] Alamgir A. N. M. (2018). Secondary metabolites: secondary metabolic products consisting of C and H. *In Therapeutic Use of Medicinal Plants and their Extracts*.

[48] Bansal V., Kumar P., Tuteja S. K., Siddiqui M. W. (2017). Diverse utilization of plant-originated secondary metabolites. *In Plant Secondary Metabolites*.

[49] Chebouat E., Gherraf N., Dadamoussa B., Allaoui M., Chirite A., Zellagui A. (2016). Chemical composition of the dichloromethane extract of *Ephedra alata* leaves and flowers. *Der Pharmacia Letter*.

[50] Kumar A., Irchhaiya R., Yadav A. (2015). Metabolites in plants and its classification. *World Journal of Pharmaceutical Sciences*.

[51] Aydoğan C. (2020). Recent advances and applications in LC-HRMS for food and plant natural products: a critical review. *Analytical and Bioanalytical Chemistry*.

[52] Ersoy E., Eroglu Ozkan E., Boga M., Mat A. (2020). Evaluation of *in vitro* biological activities of three *Hypericum* species (*H. calycinum, H. confertum,* and *H. perforatum*) from Turkey. *South African Journal of Botany*.

[53] Kepceoğlu A., Gündoğdu Y., Ledingham K. W. D., Kilic H. S. (2020). Real-Time distinguishing of the xylene isomers using photoionization and dissociation mass spectra obtained by Femtosecond Laser Mass Spectrometry (FLMS). *Analytical Letters*.

[54] Alvarez-Rivera G., Ballesteros-Vivas D., Parada-Alfonso F., Ibañez E., Cifuentes A. (2019). Recent applications of high resolution mass spectrometry for the characterization of plant natural products. *TrAC Trends in Analytical Chemistry*.

[55] Ballesteros-Vivas D., Álvarez-Rivera G., Ibáñez E., Parada-Alfonso F., Cifuentes A. (2019). A multi-analytical platform based on pressurized-liquid extraction, in vitro assays and liquid chromatography/gas chromatography coupled to high resolution mass spectrometry for food by-products valorisation. part 2: characterization of bioactive compounds from goldenberry (Physalis peruviana L.) calyx extracts using hyphenated techniques. *Journal of Chromatography A*.

[56] Puebla G. G., Iglesias A., Gómez M. A., Prámparo M. B. (2017). Fossil record of *Ephedra* in the lower cretaceous (Aptian), Argentina. *Journal of Plant Research*.

[57] Wu H., Ma Z., Wang M.-M., Qin A.-L., Ran J.-H., Wang X.-Q. (2016). A high frequency of allopolyploid speciation in the gymnospermous genusEphedraand its possible association with some biological and ecological features. *Molecular Ecology*.

[58] Hollander J. L., Vander Wall S. B., Baguley J. G. (2010). Evolution of seed dispersal in North American *Ephedra*. *Evolutionary Ecology*.

[59] Hollander J. L., Vander Wall S. B. (2009). Dispersal syndromes in North American *Ephedra*. *International Journal of Plant Sciences*.

[60] Rydin C., Pedersen K. R., Crane P. R., Friis E. M. (2006). Former diversity of Ephedra (Gnetales): evidence from early cretaceous seeds from Portugal and North America. *Annals of Botany*.

[61] Yang Y., Ferguson D. K. (2015). Macrofossil evidence unveiling evolution and ecology of early Ephedraceae. *Perspectives in Plant Ecology, Evolution and Systematics*.

[62] Pearson H..H. W. (2010). *Gnetales*.

[63] Foster A. S., Gifford E. M. (1989). *Morphology and Evolution of Vascular Plants*.

[64] Stapf O. (1889). “*Die Arten der gattung Ephedra,”* KK hof-und staatsdruckerei. *Commission bei F*.

[65] Rothwell G. W., Stockey R. A. (2013). Evolution and phylogeny of gnetophytes: evidence from the anatomically preserved seed cone *Protoephedrites eamesii* gen. et sp. nov. and the seeds of several bennettitalean species. *International Journal of Plant Sciences*.

[66] Yang Y., Wang Q. (2013). The earliest fleshy cone of *Ephedra* from the early cretaceous Yixian Formation of northeast China. *PLoS One*.

[67] Yang Y., Lin L., Ferguson D. K. (2015). Parallel evolution of leaf morphology in gnetophytes. *Organisms Diversity & Evolution*.

[68] Ferguson K., Yang Y., Lin & David L. (2015). *Org Divers Evol*.

[69] Loera I., Sosa V., Ickert-Bond S. M. (2012). Diversification in North American arid lands: niche conservatism, divergence and expansion of habitat explain speciation in the genus *Ephedra*. *Molecular Phylogenetics and Evolution*.

[70] Rydin C., Khodabandeh A., Endress P. K. (2010). The female reproductive unit of *Ephedra* (Gnetales): comparative morphology and evolutionary perspectives. *Botanical Journal of the Linnean Society*.

[71] Ickert-Bond S. M., Rydin C., Renner S. S. (2009). A fossil-calibrated relaxed clock forEphedraindicates an Oligocene age for the divergence of Asian and New World clades and Miocene dispersal into South America. *Journal of Systematics and Evolution*.

[72] Rydin C., Pedersen K. R., Friis E. M. (2004). On the evolutionary history of *Ephedra*: cretaceous fossils and extant molecules. *Proceedings of the National Academy of Sciences*.

[73] Kunzmann L., Mohr B. A., Bernardes-de-Oliveira M. E. (2009). *Cearania heterophylla* gen. nov. et sp. nov., a fossil gymnosperm with affinities to the Gnetales from the Early Cretaceous of northern Gondwana. *Review of Palaeobotany and Palynology*.

[74] Cladera G., del Fueyo G., Villar de Seoane L., Archangelsky S. (2007). Early cretaceous riparian vegetation in patagonia, Argentina. *Revista del Museo Argentino de CIencias Naturales*.

[75] Krassilov V. A. (2009). Diversity of Mesozoic gnetophytes and the first angiosperms. *Paleontological Journal*.

[76] Krassilov V. (1982). Early cretaceous flora of Mongolia. *Palaeontographica Abteilung B Palaeophytologie*.

[77] Yang Y. (2010). A review on gnetalean megafossils: problems and perspectives. *Taiwania*.

[78] Rydin C., Friis E. (2010). A new Early Cretaceous relative of Gnetales: siphonospermum simplex gen. et sp. nov. from the Yixian Formation of Northeast China. *BMC Evolutionary Biology*.

[79] Yang Y., Geng B.-Y., Dilcher D. L., Chen Z.-D., Lott T. A. (2005). Morphology and affinities of an early cretaceous Ephedra (Ephedraceae) from China. *American Journal of Botany*.

[80] Liu H. M., Ferguson D. K., Hueber F. M., Li C. S., Wang Y. F. (2008). Taxonomy and systematics of *Ephedrites cheniae* and *Alloephedra xingxuei* (Ephedraceae). *Taxon*.

[81] Meena B., Singh N., Mahar K. S., Sharma Y. K., Rana T. S. (2009). Molecular analysis of genetic diversity and population genetic structure in *Ephedra foliata*: an endemic and threatened plant species of arid and semi-arid regions of India. *Physiology and Molecular Biology of Plants*.

[82] Fuster F., Traveset A. (2019). Evidence for a double mutualistic interaction between a lizard and a Mediterranean gymnosperm, *Ephedra fragilis*. *AoB Plants*.

[83] Ickert‐Bond S. M., Renner S. S. (2016). The Gnetales: recent insights on their morphology, reproductive biology, chromosome numbers, biogeography, and divergence times. *Journal of Systematics and Evolution*.

[84] Wang X.-Q., Ran J.-H. (2014). Evolution and biogeography of gymnosperms. *Molecular Phylogenetics and Evolution*.

[85] Qin A. L., Wang M. M., Cun Y. Z. (2013). Phylogeographic evidence for a link of species divergence of *Ephedra* in the Qinghai-Tibetan Plateau and adjacent regions to the Miocene Asian aridification. *PloS One*.

[86] Ickert-Bond S. M., Wojciechowski M. F. (2004). Phylogenetic relationships in *Ephedra* (Gnetales): evidence from nuclear and chloroplast DNA sequence data. *Systematic Botany*.

[87] Crane P. R. (1996). The fossil history of the Gnetales. *International Journal of Plant Sciences*.

[88] Wang X., Zheng S. (2010). Whole fossil plants of *Ephedra* and their implications on the morphology, ecology and evolution of Ephedraceae (Gnetales). *Chinese Science Bulletin*.

[89] Yang Y. (2014). A systematic classification of Ephedraceae: living and fossil. *Phytotaxa*.

[90] Pellati F., Benvenuti S. (2008). Determination of ephedrine alkaloids in *Ephedra* natural products using HPLC on a pentafluorophenylpropyl stationary phase. *Journal of Pharmaceutical and Biomedical Analysis*.

[91] Jaradat N., Hussen F., Al Ali A. (2015). Preliminary phytochemical screening, quantitative estimation of total fl^2^avonoids, total phenols and antioxidant activity of *Ephedra alata* Decne. *Journal of Materials and Environmental Science*.

[92] Alali F. Q., Tawaha K., El-Elimat T., Syouf M. (2007). Antioxidant activity and total phenolic content of aqueous and methanolic extracts of Jordanian plants: an ICBG project. *Natural Product Research*.

[93] Nasar M. Q., Khalil A. T., Ali M., Shah M., Ayaz M., Shinwari Z. K. (2019). Phytochemical analysis, *Ephedra Procera* CA Mey. mediated green synthesis of silver nanoparticles, their cytotoxic and antimicrobial potentials. *Medicina*.

[94] Mellado M., Soto M., Madrid A. (2019). *In vitro* antioxidant and antiproliferative effect of the extracts of *Ephedra chilensis* K Presl aerial parts. *BMC Complementary and Alternative Medicine*.

[95] Al-Trad B., A Al –Qudah M., Al Zoubi M. (2018). In-vitro and in-vivo antioxidant activity of the butanolic extract from the stem of Ephedra alte. *Biomedical and Pharmacology Journal*.

[96] Hegazy A. K., Mohamed A. A., Ali S. I., Alghamdi N. M., Abdel-Rahman A. M., Al-Sobeai S. (2019). Chemical ingredients and antioxidant activities of underutilized wild fruits. *Heliyon*.

[97] Kallassy H., Fayyad-Kazan M., Makki R. (2017). Chemical composition and antioxidant, anti-inflammatory, and antiproliferative activities of Lebanese *Ephedra Campylopoda* plant. *Medical Science Monitor Basic Research*.

[98] Milman B. L. (2015). General principles of identification by mass spectrometry. *TrAC Trends in Analytical Chemistry*.

[99] Pellati F., Benvenuti S. (2008). Determination of ephedrine alkaloids in *Ephedra* natural products using HPLC on a pentafluorophenylpropyl stationary phase. *Journal of Pharmaceutical and Biomedical Analysis*.

[100] Ziani B. E. C., Heleno S. A., Bachari K. (2019). Phenolic compounds characterization by LC-DAD- ESI/MSn and bioactive properties of Thymus algeriensis Boiss. & Reut. and Ephedra alata Decne. *Food Research International*.

[101] Schäfer S., Salcher S., Seiter M. (2016). Characterization of the XIAP-inhibiting proanthocyanidin fraction of the aerial parts of *Ephedra sinica*. *Planta Medica*.

[102] Ibragic S., Sofić E. (2015). Chemical composition of various *Ephedra* species. *Bosnian Journal of Basic Medical Sciences*.

[103] Gul R., Jan S. U., Faridullah S., Sherani S., Jahan N. (2017). Preliminary phytochemical screening, quantitative analysis of alkaloids, and antioxidant activity of crude plant extracts from *Ephedra intermedia* indigenous to Balochistan. *The Scientific World Journal*.

[104] Choi S. Y., Jeong B., Jang H. S. (2019). Simultaneous analysis of four standards of the herbal formula, DF-02, of *Ephedra intermedia* and *Rheum palmatum*, using by High Performance Liquid Chromatography-Ultraviolet Detector (HPLC-UVD). *Natural Product Sciences*.

[105] Lim J., Lee H., Ahn J. (2018). The polyherbal drug GGEx18 from Laminaria japonica, Rheum palmatum, and Ephedra sinica inhibits hepatic steatosis and fibroinflammtion in high-fat diet-induced obese mice. *Journal of Ethnopharmacology*.

[106] Jeong B., Yoon Y., Shin S. S., Kwon Y. S., Yang H. (2017). Simultaneous determination of (+)-Pseudoephedrine and (-)-Ephedrine in *Ephedra intermedia* by HPLC-UV. *Korean Journal of Pharmacognosy*.

[107] Hyuga S., Hyuga M., Oshima N. (2016). Ephedrine alkaloids-free *Ephedra* herb extract: a safer alternative to *Ephedra* with comparable analgesic, anticancer, and anti-influenza activities. *Journal of Natural Medicines*.

[108] Oshima N., Maruyama T., Yamashita T. (2018). Two flavone C-glycosides as quality control markers for the manufacturing process of ephedrine alkaloids-free *Ephedra* herb Extract (EFE) as a crude drug preparation. *Journal of Natural Medicines*.

[109] Wang J.-W., Chiang M.-H., Lu C.-M., Tsai T.-H. (2016). Determination the active compounds of herbal preparation by UHPLC-MS/MS and its application on the preclinical pharmacokinetics of pure ephedrine, single herbal extract of Ephedra, and a multiple herbal preparation in rats. *Journal of Chromatography B*.

[110] Mei F., Xing X.-f., Tang Q.-f. (2016). Antipyretic and anti-asthmatic activities of traditional Chinese herb-pairs, *Ephedra* and *Gypsum*. *Chinese Journal of Integrative Medicine*.

[111] Lu N.-w., Li N., Dong Y.-m. (2015). A Rapid hydrophilic interaction liquid chromatographic method for simultaneous determination of three ephedrine alkaloids in *Ephedra* herb and its preparations. *Journal of Liquid Chromatography & Related Technologies*.

[112] Zhang D., Deng A.-J., Ma L. (2016). Phenylpropanoids from the stems of Ephedra sinica. *Journal of Asian Natural Products Research*.

[113] Shawarb N., Jaradat N., Abu-Qauod H., Alkowni R., Hussein F. (2017). Investigation of antibacterial and antioxidant activity for methanolic extract from different edible plant species in Palestine. *Moroccan Journal of Chemistry*.

[114] Jaradat N. A., Shawahna R., Eid A. M., Al-Ramahi R., Asma M. K., Zaid A. N. (2016). Herbal remedies use by breast cancer patients in the West Bank of Palestine. *Journal of Ethnopharmacology*.

[115] Khan A., Jan G., Khan A., Gul Jan F., Bahadur A., Danish M. (2017). *In vitro* antioxidant and antimicrobial activities of *Ephedra gerardiana* (root and stem) crude extract and fractions. *Evidence-Based Complementary and Alternative Medicine*.

[116] Park S. Y., Yi E. H., Kim Y., Park G. (2019). Anti-neuroinflammatory effects of *Ephedra sinica* Stapf extract-capped gold nanoparticles in microglia. *International Journal of Nanomedicine*.

[117] Gajardo S., Aguilar M., Stowhas T. (2016). Determination of sun protection factor and antioxidant properties of six Chilean *Altiplano* plants. *Boletín Latinoamericano y del Caribe de Plantas Medicinales y Aromáticas*.

[118] Palici I. F., Liktor-Busa E., Zupkó I. (2015). Study of in vitro antimicrobial and antiproliferative activities of selected Saharan plants. *Acta Biologica Hungarica*.

[119] Fazeli-Nasab B., Mousavi S. R. (2019). Antibacterial activities of *Ephedra sinica* herb extract on standard and clinical strains of *Pseudomonas aeruginosa*. *Journal of Medical Bacteriology*.

[120] Mahmood N., Nazir R., Khan M. (2019). Phytochemical screening, antibacterial activity and heavy metal analysis of ethnomedicinal recipes and their sources used against infectious diseases. *Plants*.

[121] Ghanem S., El-Magly U. I. (2008). Antimicrobial activity and tentative identification of active compounds from the medicinal *Ephedra alata* male plant. *Journal of Taibah University Medical Sciences*.

[122] Al-Awaida W., Al-Hourani B. J., Akash M. (2018). *In vitro* anticancer, anti-inflammatory, and antioxidant potentials of *Ephedra aphylla*. *Journal of Cancer Research and Therapeutics*.

[123] Hoshyar R., Mostafavinia S. E., Zarban A. (2015). Correlation of anticancer effects of 12 Iranian herbs on human breast adenocarcinoma cells with antioxidant properties. *Free Radicals & Antioxidants*.

[124] Mohammad S., Masoumeh H., Gholamali J., Hoda T. (2017). Ephedraceae as a treatment for hyperlipidemia and hyperglycemia: an experimental study. *Journal of Autoimmune Disorders*.

[125] Uttra A. M. (2017). Assessment of anti-arthritic potential of *Ephedra gerardiana* by in vitro and in vivo methods. *Bangladesh Journal of Pharmacology*.

[126] Lee S. E., Lim C., Lim S., Lee B., Cho S. (2019). Effect of *Ephedra* herb methanol extract on high-fat diet-induced hyperlipidaemic mice. *Pharmaceutical Biology*.

[127] Nakamori S., Takahashi J., Hyuga S. (2019). “Analgesic effects of *Ephedra* herb extract, ephedrine alkaloids–free *Ephedra* herb extract, ephedrine, and pseudoephedrine on formalin-induced Pain. *Biological and Pharmaceutical Bulletin*.

[128] Oshima N., Yamashita T., Hyuga S. (2016). Efficiently prepared ephedrine alkaloids-free *Ephedra* Herb extract: a putative marker and antiproliferative effects. *Journal of Natural Medicines*.

[129] Lin Y. C., Chang C. W., Wu C. R. (2016). Antitussive, anti-pyretic and toxicological evaluation of Ma-Xing-Gan-Shi-Tang in rodents. *BMC Complementary and Alternative Medicine*.

[130] Lee A. Y., Jang Y., Hong S. H. (2017). Ephedrine-induced mitophagy via oxidative stress in human hepatic stellate cells. *The Journal of Toxicological Sciences*.

[131] Powell T., Hsu F. F., Turk J., Hruska K. (1998). Ma-huang strikes again: ephedrine nephrolithiasis. *American Journal of Kidney Diseases*.

[132] Nauffal M., Gabardi S. (2016). Nephrotoxicity of natural products. *Blood Purification*.

[133] Lee M. K., Cheng B. W. H., Che C. T., Hsieh D. P. H. (2000). Cytotoxicity assessment of Ma-huang (*Ephedra*) under different conditions of preparation. *Toxicological Sciences*.

[134] Wu Z., Kong X., Zhang T., Ye J., Fang Z., Yang X. (2014). Pseudoephedrine/ephedrine shows potent anti-inflammatory activity against TNF-*α*-mediated acute liver failure induced by lipopolysaccharide/d-galactosamine. *European Journal of Pharmacology*.

[135] Zheng E., Navarro V. (2016). Liver injury due to herbal and dietary supplements: a review of individual ingredients. *Clinical Liver Disease*.

[136] Mladěnka P., Patočka L. A. J. (2018). Comprehensive review of cardiovascular toxicity of drugs and related agents. *Medicinal Research Reviews*.

[137] Hudson A., Lopez E., Almalki A. J., Roe A. L., Calderón A. I. (2018). A review of the toxicity of compounds found in herbal dietary supplements. *Planta Medica*.

